# Combined endoscopic management of a postoperative rectocutaneous fistula after cystectomy using modified percutaneous endofistular and transanal endoluminal vacuum therapy

**DOI:** 10.1055/a-2344-7445

**Published:** 2024-07-03

**Authors:** Ahmed Alwali, Clemens Schafmayer, Imad Kamaleddine

**Affiliations:** 1 39071 Department of General, Visceral, Thoracic, Vascular and Transplant Surgery, Rostock University Medical Center, Rostock, Germany

A 79-year-old man presented with a transanal purulent discharge following cystectomy with rectal injury 10 months previously. At that time, surgical closure with suturing of the rectal defect had been performed, along with the creation of a colostomy. Endoscopic examination revealed a perforation 5 cm from the anal verge. An attempt was made to close the perforation with an over-the-scope clip; however, during the follow-up endoscopy, a significant defect was observed in rectum, with an accompanying large cavity. The patient then underwent 6 weeks of inpatient therapy with endoluminal transanal vacuum therapy, which resulted in a reduction of the cavity size. The patient returned to us 4 months later with persistent purulent discharge from the caudal pole of the laparotomy scar and anus ( Fig. 1Fig. 1 ). Endoscopic examination revealed a defect in the anterior wall of the rectum 5 cm from the anal verge, with a cavity full of pus and a rectocutaneous fistula. We opted for a combined approach involving endoluminal and endofistular vacuum therapy for 3 weeks in the hospital setting ( Fig. 2Fig. 2 ).

**Fig. 1:**
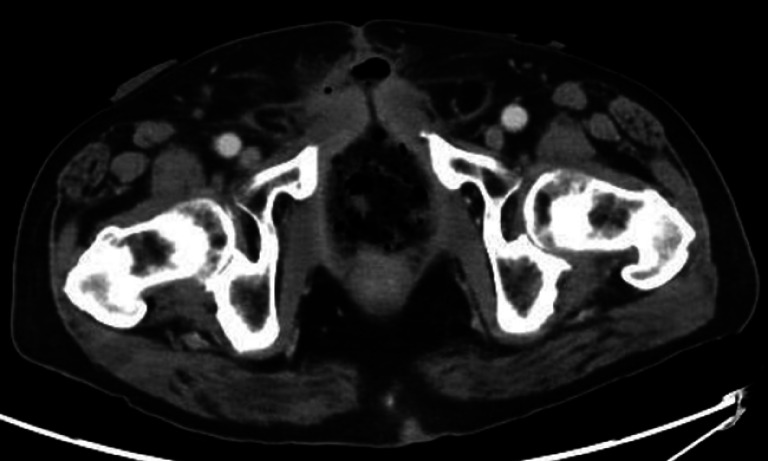
**Fig. 1** A computed tomography image showing a pararectal cavity with a complex rectocutaneous fistula.

**Fig. 2:**
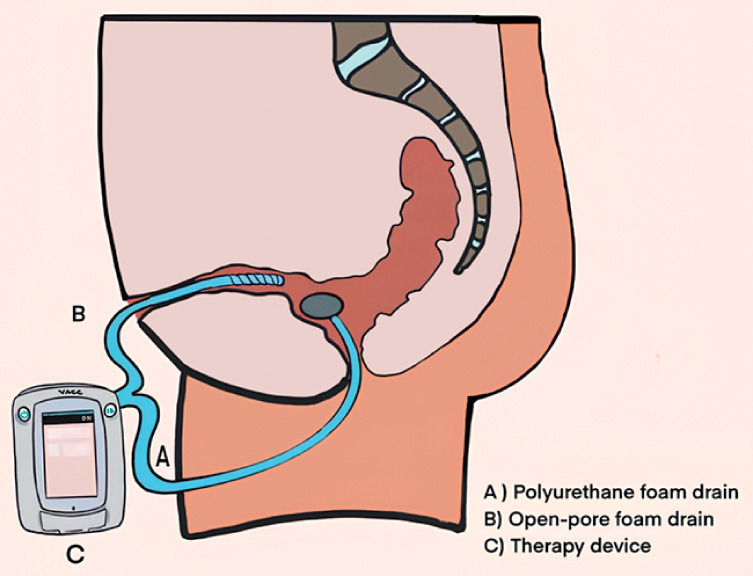
**Fig. 2** Schematic of the combined modified percutaneous endofistular and transanal endoluminal vacuum therapy.

Endoscopic vacuum therapy was initiated using an open-pore polyurethane foam drain (EndoSponge; B. Braun, Melsungen, Germany) placed transanally into the cavity, along with a customized open-pore film drain (Suprasorb CNP; Lohmann & Rauscher, Rengsdorf, Germany) inserted through the skin ( Fig. 3Fig. 3 ) 11 . Suction of –125  mmHg was applied (ACTIV.A.C; KCI, San Antonio, Texas, USA) ( Video 1Video 1 ).

**Fig. 3:**
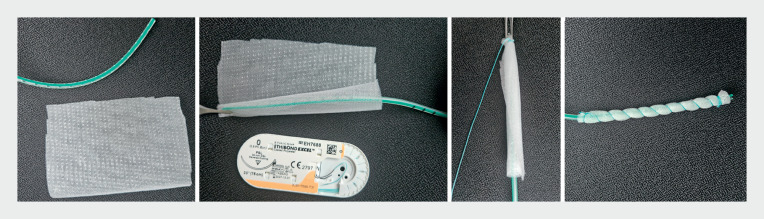
**Fig. 3** Photographs showing the creation of an open-pore film drain, which involves wrapping a strip of double-layered open-pore film around a small drainage tube and securing it in place with a suture.

The combined endofistular and endoluminal endoscopic management of a postoperative complex rectocutaneous fistula after cystectomy.Video 1Video 1

Owing to the patientʼs preference for outpatient therapy, we continued therapy with outpatient percutaneous endofistular vacuum therapy, as previously described, changed twice weekly for an additional 3 weeks, progressively shortening the drain length with each adjustment. Upon follow-up examination 3 days later, the fistula had completely closed, and the pararectal cavity could no longer be visualized.

Endofistular vacuum therapy represents an effective method for the management of complex rectocutaneous fistulas, serving as a valuable adjunctive technique in the treatment of challenging cases.

Endoscopy_UCTN_Code_TTT_1AQ_2AG

## Notes

Endoscopy E-Videos https://eref.thieme.de/e-videos
*E-Videos* is an open access online section of the journal *Endoscopy* , reporting on interesting cases and new techniques in gastroenterological endoscopy. All papers include a high-quality video and are published with a Creative Commons CC-BY license. Endoscopy E-Videos qualify for HINARI discounts and waivers and eligibility is automatically checked during the submission process. We grant 100% waivers to articles whose corresponding authors are based in Group A countries and 50% waivers to those who are based in Group B countries as classified by Research4Life (see: https://www.research4life.org/access/eligibility/ ). This section has its own submission website at https://mc.manuscriptcentral.com/e-videos .
